# A Case of Tetanus, Which Terminated in Recovery

**Published:** 1807-03

**Authors:** P. Tracy

**Affiliations:** Norwich, in Connecticut


### A Case *of* Tetanus, *which terminated in Recovery.* *By Dr.* P. Tracy, *of Norwich, in Connecticut.*

AMONG the variety of diseases incident to mankind, few are involved in more obscurity as to their pathology, and perhaps no one has more generally baffled the skill of the physician and proved fatal, than cases of tetanus. The insurmountable difficulties that occur in attempting to investigate the principles on which the regular operation of the nervous power productive of muscular motion depends, renders it peculiarly embarrassing to explain the phenomena of diseases immediately implicating this intricate part of the animal system; these difficulties have appeared so great to Dr. Cullen and other writers of eminence, that in treating of tetanus, a disease confessedly belonging to this head, they have omitted all attempts to explain the pathology of the disorder, and candidly avowed their ignorance on this subject. The doctrine of sympathy, so ingeniously advocated by the late Dr. Darwin, has unfolded many curious facts; but although his discoveries have expanded the field of medical science, and promise a rich harvest to those who have abilities and industry sufficient to explore the path he has pointed out; still, at present, many wonderful operations connected with animal life remain involved in obscurity, notwithstanding the light he has really shed on some part of the subject. With due deference to his elevated capacity and superior ingenuity, I will venture to say that his explanation of the physical causes of the spasmodic or convulsive form of disorders (including tetanus), at least is involved in intricacy and doubt.

I feel no way disposed to attempt an explanation of the pathology of a disease thus left enveloped in obscurity and doubt by the first medical characters, and am conscious of being in every respect unqualified for the task. This disorder, like every other species which usually proves fatal, has excited the attention of physicians, and led to the adoption of a diversified round of medicaments, which, by frequently proving unsuccessful, has kept in obscurity not only the *rationale* of the disorder, but lessened the confidence of the faculty relative to the proper practice which ought to be adopted in the case.

The two varieties of the disorder marked by systematic writers, are those which arise from a puncture or *lesion* of a nerve, or that which takes place without any such exciting citing cause, but which has followed an exposure to take a violent cold. The latter of these I conceive is only entitled to the appellation of an *idiopathic tetanus,* which I believe has more generally yielded to appropriate remedies than the former; for that depending on local injury not frequently discoverable by inspection or removeable by topical application, continues, notwithstanding the utmost efforts of medical skill, to increase its effects on the nervous system, until life is exhausted and nature sinks. Under which head the case of tetanus annexed properly belongs, I confess myself at a loss to determine. How far the continued application of cold water to ulcerous legs, by varying the secretion from the sores, or by checking a morbific discharge, perhaps necessary to the animal economy, was capable of producing the complaint; or whether the effects of cold partially applied, by its general operation on the system as a debilitating power, by interrupting the native train of healthy actions, might have led to the effect; both remain questions which I am unable to decide. These doubts were the cause of much embarrassment to me in attending on the case. The happy issue of the disorder, while it affords me the highest satisfaction as regards the feelings of humanity, I judge will be deemed a sufficient apology for making the case public.

Sunday, June 1st, 1805, was called to visit J. B. a man aged forty-four years, of a robust habit, but labouring under an ulcerous state of the legs, arising from a syphilitic complaint, for which he had been using a solution of Corr. Subl. topically. I found that three days previous to my being called, after having waded in the water for some hours, for the purpose of collecting rockweed, a severe chill ensued, followed by a violent pain through the sternum, together with a stiffness and rigidity of the muscles of the neck, attended with great difficulty in opening the mouth. He had called in a neighbouring physician, and used some medicines, which afforded no relief. On examining the case, I found the subject greatly distressed with severe pain through the lower part of the sternum, and shooting from thence through the shoulders to the back part of the neck, producing a considerable retraction of the head, and his jaw so closed as to render it very difficult to get the handle of a spoon (flat-ways) between his teeth. The pain at the sternum, from thence through the back of the neck, recurred more severely at intervals, but the stricture on the jaws continued uniform and fixed. At the paroxysms of pain his sufferings were exquisite, exquisite, a universal rigidity of the dorsal muscles occurred, with a strong retraction of the head, assuming the marks of a true opisthotonos. At the termination of the severe fits of pain, a universal profuse sweat would issue forth, and for a few moments he would sink, and seem ready to expire, and then gradually revive. These severe turns would generally recur in fifteen or twenty minutes. The pulses were small and weak, and no febrile heat existed. Convinced that the case was tetanic, I applied a large epispastic to the gastric region, and directed to embrocate the neck and muscles of the spine with volatile liniment, together with the addition of oil of amber. Finding that he had been costive for some days, I directed a common enema to open the bowels, and gave him ten grains of calomel combined with a little thin mucilage, through a goose quill, which was the method he adopted to take his drinks, which, fortunately by the loss of some of his fore teeth, he was enabled to receive into his mouth. In conjunction with these medicaments, I directed him to take forty-five drops of laudanum every four hours, and repeat it at shorter intervals in case the spasms recurred severely. To the ulcers on the legs I directed the application of flax-seed cataplasms, and draughts on his feet. For support, I recommended veal broth and the use of Madeira wine.

Monday, June 2d. On visiting the patient, found that he had rested some during the night, but that he was still subject to pains as the day before. The pain at the sternum had somewhat abated, the blister having fully drawn. The jaws remained fixed as the day before. The laudanum had been continued with regularity, and the enema had produced some alvine discharge. The pulse remained low, without febrile heat. I directed the use of laudanum to be continued as before prescribed, and repeated the same quantity of ten gtains of calomel, with directions to continue the use of the liniment to the spine and throat, and the support with the wine and broth to be continued as usual.

Tuesday, June 3d. On visiting the patient, found that he had passed a distressed night. The jaw remained immoveable and closed, and the spasms had recurred with great violence, so as to lead his attendants to apprehend that his hip joints were dislocated, attended with great rigidity of the dorsal muscles, and a constant retraction of the head. He made some efforts, during the night, with his hands to open his mouth, but found them unavailing, vailing, and compared the impossibility to effect it with that of severing a billet of wood lengthways by manual force. At periods the power of swallowing was wholly lost, and then in a short time would recur; but he always appeared greatly distressed in the act. I directed the laudanum to be increased to sixty drops every four hours, and to take twenty-five drops of the ol. succin. at a dose once in two or three hours, as the violence of the spasms might require, also to continue the liniment to the spine, &c. and repeated ten grains of calomel, and ordered him to take wine with freedom.

Wednesday, June 4th. On visiting the patient, found that he had passed a distressed night. The jaws continued closed. The spasms had recurred with great severity, and had extended to the lower extremities, which frequently were extremely convulsed, and produced the most exquisite distress, with a general rigidity of the dorsal muscles, which rendered the spine and lower limbs perfectly inflexible, and kept the head drawn back. He swallowed with difficulty, but had been able to take the laudanum as directed. His pulse remained low, and his breathing at times was laborious with dyspnœa. I directed the continuation of laudanum and ol. succin. in the usual quantity, and repeated ten grains of calomel, and ordered two drachms of strong mercurial ointment to be rubbed on the thighs, and the support to be continued with the wine freely. On inspecting the ulcers on the legs I found them to assume a flaccid appearance, with a sanious discharge issuing from them, and one of considerable depth on the gastrocnemius muscle appeared of a livid hue. For these I recommended a strong decoction of the bark to wash the ulcers, and dressed them with lint wet with the tincture of myrrh, and covered them with pledgets spread with ungt. Basilic.

Thursday, June 5th. The patient continued much as yesterday. He had taken the laudanum and ol. succin. as directed, and the mercurial ointment had been rubbed on the thighs. He had slept some during the night, but was subject to frequent startings, which were always productive of severe spasms. The masseter muscle still kept firmly contracted. When he slept, an evident relaxation was observed to take place. The pain through the sternum had considerably abated, and his lower limbs seemed most affected, and at times were exceedingly convulsed. Continued the dressings to the sores, which had assumed a better appearance; their margin appeared less livid, and the matter discharged became more healthy. The laudanum num was directed to be continued as yesterday, and the same quantity of mercurial ointment was repeated on the thighs.

Friday, June 6th. On visiting the patient, found him much as yesterday, except the retraction of the head had in some degree abated, with less pain in that region. The mouth still continued obstinately closed; when sleeping it considerably relaxed. The severe cramps on the extreme parts still continued to occur, and extended equally to the arms and lower limbs. He had slept some during the night, but was aroused by the smallest noise, and always awoke in great distress. The calomel he had before taken during the night had griped him severely, and been productive, for the first time, of several powerful discharges by the bowels, which had much weakened him. The ulcers discharged a more laudable pus. Their margin had in a considerable degree lost their flaccid appearance, and assumed a more florid hue. I directed the continuation of the laudanum and ol. succin. in the same quantity, and at the same intervals as yesterday, and ordered two additioal drachms of mercurial ointment to be rubbed on the thighs, and to supply him as freely with wine as he could bear without heating him too much.

Saturday, June 7th. On visiting the patient, found that he had passed a tolerable night, though subject to frequent startings and severe crampy affections in the extreme parts. The jaws at times when awake, seemed to be a little relaxed, with less rigidity of the muscles of the neck and drawing back of the head. He still remained incapable of taking food, except through a tube made for that pupose. For the first time some effect of the mercury appeared on the mouth, discoverable by the odour in the breath and the flowing of the saliva. Directed the continuation of laudanum in the usual quantity, and the repetition of the mercurial ointment by rubbing, with orders to continue the wine with freedom. The ulcers had assumed a bright appearance, and discharged good pus.

Sunday, June 8th. The patient continued much as yesterday; had slept some during the night, but was subject to frequent startings and severe crampy affections, principally in the lower extremities. The jaws remained still so closed as to render him incapable of taking sustenance, except through the tube. A flowing of saliva still continued, with soreness of the mouth, fully evincive of the effects of the mercury. He had taken the laudanum as as yesterday, together with considerable quantity of wine. Directed the wine and laudanum to be continued as usual.

Monday, June 9th. The patient passed a very distressed night. The crampy affections of the lower limbs recurred with great severity, and produced the most exquisite distress. His jaws remained firmly closed. An unusual rigidity of the muscles existed so as to render him wholly inflexible. The mercury continued to affect the mouth. Had a spontaneous discharge by stool in the early part of the day. Ordered the laudanum increased to eighty drops once in four hours, and the wine to be used freely; the embrocation to the spine and limbs to be continued. The sores appeared well.

Tuesday, June 10th. He had rested more the last night than usual. The cramps, though still severe, had been less frequent and violent than ordinary. A rashy efflorescence had appeared on the breast, shoulders, &c. The mouth still evinced the operation of mercury on the system. Directed the continuation of eighty drops of laudanum every four hours; and, anxious to support the mercurial action, directed a drachm of the ointment to be rubbed on in the usual manner. A free use of the wine was directed to be continued.

Wednesday, June 11th. On visiting the patient, found that he had passed a more comfortable night. The spasms had less frequently recurred, and the rigidity of the muscles of the neck and spine had considerably lessened, although the jaws continued fixed as usual. The rashy efflorescence still continued on the surface, and two or three small imposthumations appeared forming on the legs. A gentle salivation continued. I directed a continuation of eighty drops of the laudanum every four hours. Still wishing to actively support the mercurial process, ordered one drachm of the mercurial unguent to be repeated, and wine to be continued as usual with freedom.

Thursday, June 12th. The patient to day appeared more placid; had rested tolerably during the night. He had been able once or twice during the night to voluntarily open his mouth further than before. This faculty, however, was but momentary. The general rigidity of the muscles had much abated. The efflorescence still continued, and the imposthumations seemed advanciug towards suppuration. The mouth had become very sore, with a free discharge of saliva. I directed a continuation of eighty drops of laudanum at the usual periods. As he had been costive for some days, I directed a strong infusion fusion of senna, sweetened with manna, to be given him, and the free use of the wine to be continued.

Friday, June 13th. The patient had passed a comfortable night; the spasms had much abated. The physic had produced two free dejections. The stricture of the mouth still continued considerable. A gentle salivation also continued, and the efflorescence remained on the surface as usual. Directed the usual quantity of laudanum and the free use of wine.

Saturday, June 14th. The patient still continued comfortable, although very weak. The spasms had mostly subsided. He had slept quietly. Directed the diminution of the laudanum to sixty drops every four hours, and the support to be continued.

Sunday, June 15th. The patient much as yesterday; the gentle salivation continued, and the sores on the legs appeared to be fast healing. He still continued unable to open his mouth to take food. Although the general tetanic appearance had left him, I directed a continuation of fifty drops of laudanum every six hours, and ordered his jaws to be embrocated two or three times daily with ol. olivarum camphorated.

Monday, Tuesday, Wednesday, and Thursday, June 16th, 17th, 18th, and 19th. The patient continued to generally amend, the motion of the jaws gradually increased, and the general spasmodic state seemed to have subsided. A universal eruption on the surface continued, and several small imposthumations had suppurated. The salivation still continued gently. The use of the laudanum was continued, and occasionally a small quantity of the mercurial ointment was rubbed in to support the salivation, and physic was given to keep the bowels soluble. The wine was continued with freedom. From this time the patient continued daily, although gradually, to grow better, and, at the end of three weeks from this period, was able to go abroad, and at present is apparently in a comfortable state of health.

A question will naturally arise in contemplating this case as to the relative effects of mercury and opium in effecting the cure. Opium, from its known powers as an antispasmodic, has universally been resorted to in cases of tetanus, and many instances are on the records of medicine, of cures being effected wholly by it, when given in large quantities and long persisted in; and I conceive that no prudent physician would omit its use in a case of this kind, and wholly rely on any other medicine; still many auxiliary means are justifiable to connect with its use; one of which I conceive to be an active use of mercury. This, from its known powers on the human frame, is proved to be fully capable of exciting a new general train of actions in the animal system, frequently very powerful in removing pre-existing diseases of a formidable nature; and this was the motive which induced me to adopt it in this case. How far it may be thought that the syphilitic state of the patient had any immediate connection with the tetanus, I am at loss to determine; but at least I conceive, under that circumstance, that the mercury was more clearly indicated and justifiable to adopt, and I am inclined to think, was an active agent in the cure.

